# Nano-strategies targeting cancer-associated fibroblasts to enhance immunotherapy and reverse resistance

**DOI:** 10.3389/fimmu.2025.1668199

**Published:** 2025-09-09

**Authors:** Juqing Xu, Ze Chen, Yi Qin, Lu Tan, Shaohui Xu

**Affiliations:** ^1^ Department of Hematology and Oncology, Geriatric Hospital of Nanjing Medical University, Jiangsu Province Geriatric Institute, Jiangsu Province Official Hospital, Nanjing, Jiangsu, China; ^2^ College of Pharmaceutical Science, Soochow University, Suzhou, Jiangsu, China; ^3^ Clinical Pharmacology Research Lab, Department of Pharmacy, The First Affiliated Hospital of Soochow University, Suzhou, Jiangsu, China; ^4^ Institute of Neuroscience & Jiangsu Key Laboratory of Neuropsychiatric Diseases, Soochow University, Suzhou, Jiangsu, China; ^5^ Institute of Functional Nano & Soft Materials (FUNSOM), Soochow University, Suzhou, Jiangsu, China

**Keywords:** cancer-associated fibroblasts (CAFs), tumor microenvironment (TME), immunotherapy resistance, nanoparticle delivery systems, CAFs-targeted immunomodulation

## Abstract

Cancer-associated fibroblasts (CAFs) are significant contributors to the establishment of the immunosuppressive tumor microenvironment (TME) and pose a significant challenge to the effectiveness of successful immunotherapy. CAFs can secrete cytokines, chemokines, and extracellular matrix components; inhibit the invasion of immune cells; promote regulatory cell populations; and induce T cell exclusion phenotypes, thereby lowering the effectiveness of immune checkpoint inhibitors (ICIs). With the development of the field of nanotechnology, increasing studies have paid attention to employing nano-strategies to specifically control and target CAFs. These nanoplatforms can transport therapeutic cargos, e.g., CAF-toxic chemicals, signal regulators, or phenotype-modifying agents, precisely to CAFs, respectively, lowering systemic toxicity. Furthermore, the combination therapy of CAF-targeting nanoparticles and immune checkpoint inhibitors had, in preclinical scenarios, the synergistic effect of promoting T cell infiltration, antigen presentation, and cytotoxicity. However, heterotypic CAF subpopulations, inconsistency of different cancer models, inefficient cargo delivery, and translatability constraints in the clinic are serious challenges. Development of multifunctional and stimulus-active nanomedicine has great potential to overcome these challenges. Initial clinical trials, including fibroblast activation protein (FAP)-targeted CAR-T cells and antibody-drug conjugates, highlight the increasing translational potential of CAF-targeted nano-immunotherapy. This review summarizes the current progress in CAF-targeted nano-immunotherapy, emphasizing that a comprehensive molecular understanding and thorough clinical validation are essential for facilitating its clinical application in the treatment of solid malignancies.

## 1 Introduction

Immunotherapy has transformed cancer therapy, providing durable tumour response and greatly enhancing survival in some patients (1, 2). Nonetheless, it becomes ineffective as a result of the immunosuppressive tumor immune microenvironment (TIME) as well as complicated immune evasion methods, for various persons (3–6). Immunosuppression by cytokine production, extracellular matrix remodeling, and physical expulsion of immune cells by CAFs, the most dominant stromal element of TIME (7, 8). CAFs obstruct immune cell infiltration, inhibit T cell function, and enhance the expression of immunological checkpoints, thereby diminishing therapeutic responses and encouraging treatment resistance. Targeting CAFs has consequently emerged as a possible technique to enhance the efficacy of immune therapy. Targeting CAFs has thus emerged as a promising strategy to augment immunotherapy efficacy (9–12). Nanotechnology demonstrates advantages in targeting CAFs, facilitating the accurate delivery and targeted modulation of CAFs to enhance therapeutic drug accumulation and specificity within the tumor microenvironment. Utilizing stimuli-responsive nanocarriers enables the targeted release of drugs at disease sites, thereby reducing off-target effects and strengthening the remodeling of the TIME, ultimately improving therapy outcomes. This review highlights recent progress in nanotechnology methods targeting CAFs, focusing on their contributions to enhancing immunotherapy, overcoming therapy resistance, and regulating the TME. Moreover, significant potential and challenges are discussed to provide insights for future translational research.

## 2 CAFs and their role in immunotherapy resistance

### 2.1 Origin and heterogeneity of CAFs

**Figure 1:**
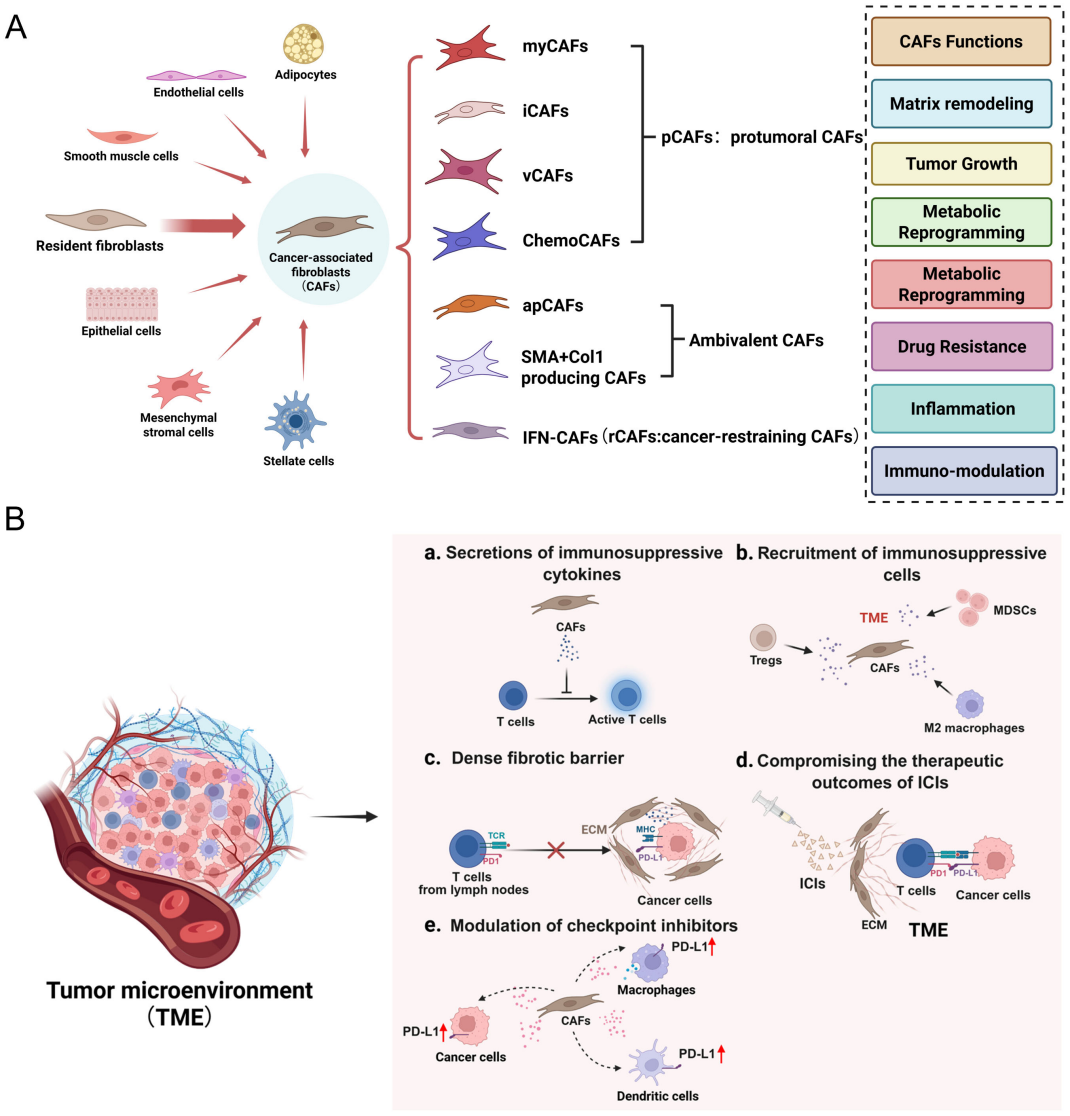
**(A)** The origin, subtypes, and functions of CAFs in TME. **(B)** CAFs induce immunosuppressive TME through distinct mechanisms.

CAFs are a highly diverse and heterogeneous group of cells with complex and varied sources (**Figure 1A**). The majority of CAFs originate from tissue-resident fibroblasts, but there are also some CAFs arising from pericytes, mesenchymal stem cells, and other precursors when tumor-derived signals are activated (13–15). This cellular diversity endows CAFs with noticeable phenotypic and functional heterogeneity, complicating their roles in tumor progression and immune regulation. Classified by function, CAFs can be divided into subtypes such as immunosuppressive CAFs (iCAFs), matrix-generating CAFs (myCAFs), and antigen-presenting CAFs (apCAFs) (16, 17). It is worth noting that CAFs have phenotypic plasticity and can dynamically shift between states induced by cues from TME, thus playing diverse roles in tumor progression and immune modulation (18, 19).

### 2.2 CAF-Mediated Immune Suppression

CAFs contribute to immunosuppression in the TME by secreting immunomodulatory cytokines, remodeling the extracellular matrix, and limiting immune cell infiltration (**Figure 1B**). CAFs actively reshape the tumor immune microenvironment by secreting immunomodulatory cytokines, including TGF-β, IL-6, and CXCL12. These factors increase T cell exhaustion, promote the expansion of regulatory T cells (Tregs), and reduce effector T cell infiltration, collectively dampening antitumor immunity. In addition, CAF-derived chemokines, including CCL2 and CXCL12, recruit Tregs and myeloid-derived suppressor cells (MDSCs), thereby reinforcing the immunosuppressive milieu (20, 21). Concurrently, CAFs contribute to extracellular matrix (ECM) remodeling by depositing collagen and fibronectin, thereby generating a dense fibrotic barrier that physically restricts T cell access to tumor cores, thereby facilitating immune evasion (22–24).

Among the heterogeneous CAF subpopulations, iCAFs, myCAFs, and apCAFs contribute differently to the immunosuppressive tumor microenvironment. iCAFs (inflammatory CAFs) are characterized by high secretion of cytokines such as IL-6, CXCL12, and TGF-β, which promote the recruitment of immunosuppressive cells like Tregs and MDSCs, and inhibit effector T cell function. myCAFs (myofibroblastic CAFs) primarily produce extracellular matrix components including collagen and fibronectin, leading to physical exclusion of T cells from the tumor core and contributing to desmoplasia. In contrast, apCAFs (antigen-presenting CAFs) express MHC class II molecules and are capable of presenting antigens to CD4+ T cells, but lack co-stimulatory molecules, thereby inducing T cell anergy or tolerance rather than activation. Together, these distinct subtypes synergize to construct an immune-excluded, immunosuppressive microenvironment that impairs effective antitumor immunity.

### 2.3 CAFs and therapy resistance

CAFs play a crucial role in mediating resistance to immune checkpoint inhibitors, particularly PD-1/PD-L1 blockade. CAFs enhance immunosuppressive signaling by producing TGF-β and other suppressive cytokines, thereby suppressing T cell activation and limiting their infiltration into tumor regions (25–28). CAFs have been shown to upregulate PD-L1 expression on both tumor and immune cells, thereby diminishing the efficacy of PD-1/PD-L1 blockade therapy (29, 30). As a result, tumors enriched with CAFs tend to exhibit resistance to immune checkpoint inhibition. Moreover, CAF-induced ECM reconstruction and cytokine release result in a T-cell exclusion phenotype, in which effector T cells localize at the tumor edge but are unable to penetrate the tumor interior—a major contributor to immunotherapy failure (31).

## 3 Nanoparticle-based strategies for CAF modulation

### 3.1 Nano-strategies for targeting CAFs

#### 3.1.1 Overview and classification of nanotechnology in CAF-targeted therapy

Nanotechnology has been created as a disruptive platform for the modulation of the tumor microenvironment and targeted drug delivery. In CAF-targeted therapy, nanoparticles have numerous advantages over traditional methods, including enhanced bioavailability, elongated circulation time, stimulus-responsive release, and spatial confinement within the fibrotic tumor stroma. A variety of nanoplatforms (liposomes, micelles, dendrimers, polymeric nanoparticles, and inorganic nanocarriers) have been designed to deliver small molecules, nucleic acids, or immunomodulatory agents. Active targeting has been applied on these nanosystems through the surface modification by CAF-specific ligands (e.g., FAP antibody, PDGFR-β-binding peptides), which enable selective accumulation in CAF-rich regions. Furthermore, stimulus-responsive nanocarriers (pH, redox potential, or enzyme-activatable) enable on-demand release in the tumor microenvironment. Nanotechnology is equally competent in offering combinatorial delivery of therapeutic agents (e.g., CAF-modulators and immune checkpoint inhibitors) in a single carrier, potentially elevating therapeutic synergy. Such attributes make nanotechnology a multifaceted and effective tool in overcoming CAFs’ biophysical and immunological barriers in solid tumors.

Nanoparticles, with sizes ranging from 1 to 100 nm, exhibit unique physicochemical properties that differ markedly from those of their bulk materials, which is mainly due to their high surface area-to-volume ratio. Owing to their unique physicochemical properties, these materials have been widespread used in fields such as biomedicine, electronics, energy, and environmental science (32, 33). Classification is typically based on composition, structure, or intended function. The following are several commonly used classification approaches.

**Figure 2:**
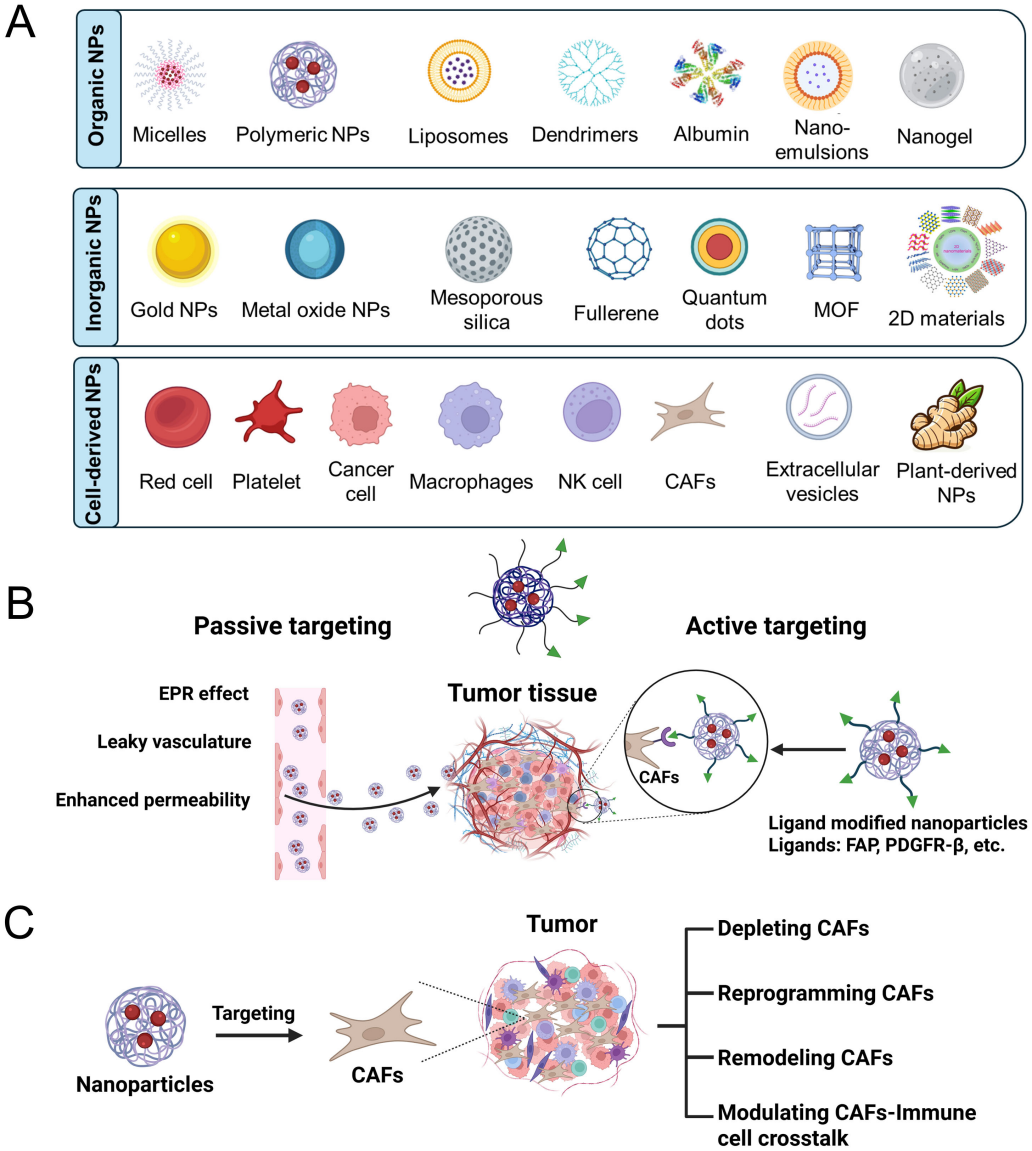
**(A)** Classification of nanoparticles by their compositional components. **(B)** Passive and active targeting CAFs by nano-strategies. **(C)** Modulation of CAFs by nanoparticles.

Based on their composition, nanoparticles are generally divided into three categories: organic, inorganic, and those derived from biological cells (**Figure 2A**). The organic nanoparticles mainly include micelles, albumin nanoparticles, liposomes, polymeric nanoparticles, dendrimers, nanoemulsions, and nanogels. Inorganic nanoparticles mainly include metal nanoparticles (e.g., Au NPs, Ag NPs), metal oxide nanoparticles (e.g., Fe_3_O_4_ NPs, TiO_2_ NPs), silica nanoparticles (e.g., SiO_2_ NPs), carbon-based nanoparticles (e.g., carbon nanotubes, graphene, carbon quantum dots), and two-dimensional (2D) nanosheets (e.g., transition metal dichalcogenides such as XS_2_ and XSe_2_, and black phosphorus). Cell-derived nanoparticles are primarily composed of membrane vesicles originating from sources such as tumor cells, macrophages, red blood cells, and platelets, including exosomes secreted by animal or plant cells.

Based on their structures, nanoparticles can be classified into four types: zero-dimensional (0D), one-dimensional (1D), two-dimensional (2D), and three-dimensional (3D). 0D nanoparticles include quantum dots and nanodots. 1D nanoparticles include nanowires, nanorods, and nanotubes. 2D nanoparticles consist of materials such as graphene, MXenes, and black phosphorus nanosheets. 3D nanoparticles include porous nanostructures, core-shell structures, and nanogels.

Nanoparticles can be divided into various types based on their stimuli-responsive properties, such as pH-responsive, temperature-responsive, light-responsive, reactive oxygen species (ROS)-responsive, and enzyme-responsive nanoparticles.

#### 3.1.2 Passive and active targeting of CAFs

Passive targeting of CAFs can be achieved with the aid of the enhanced permeability and retention (EPR) effect, which supports nanoparticles accumulating in tumor regions with hyperpermeable vasculature (**Figure 2B**) (34, 35). Due to their abundant presence in the tumor stroma, CAFs are more likely to encounter elevated concentrations of nanoparticles compared to other stromal cells. Such passive accumulation enables the possibility of regulating CAF activity and preferential delivery of therapeutic agents to the TME (36). However, the diverse and poor perfusion characteristics of the tumor stroma can limit the permeation of nanoparticles, thereby reducing delivery efficiency to CAFs and impairing therapeutic efficacy (37, 38).

Active targeting of CAFs advances beyond the EPR effect by introducing surface ligand modifications on nanoparticles to enhance targeting ability and retention within the tumor stroma (**Figure 2C**) (39, 40). As FAP, platelet-derived growth factor receptor β (PDGFRβ), and integrins are highly overexpressed on CAFs in various solid tumors, making it a promising target for selective drug delivery (41). Antibodies or peptide ligands with high affinity for these receptors can be used to decorate nanoparticles, thereby achieving selective binding to CAFs through EPR effects (42–44). This dual-targeting approach enhances cellular uptake and therapeutic precision towards CAFs, partially addressing the limitations of passive targeting delivery. Nevertheless, challenges still exist due to the heterogeneity of CAFs and side effects from possible off-target interactions with other stromal or normal fibroblastic cells.

### 3.2 CAF depletion strategies

CAF depletion nano-strategies often involve the delivery of cytotoxic agents or nucleic acid drugs (such as pDNA, siRNA, mRNA) selectively targeting CAF-associated markers such as FAP or alpha-smooth muscle actin (α-SMA) (45, 46). These strategies enable the specific removal of CAFs or the knockdown of CAF genes, thereby reducing their immunosuppressive effect and reshaping the TME to facilitate immune infiltration. These targeted approaches exert precise effects on CAFs, thus minimizing systemic toxicity and optimizing therapeutic outcomes. However, the total depletion of CAFs may pose potential risks due to their functional heterogeneity, as specific CAF subtypes may either restrain or promote tumor growth (47). Subtype-agnostic targeting strategies can disturb stromal homeostasis, which may lead to augmented tumor invasiveness and impaired therapeutic responses.

### 3.3 CAF-reprogramming approaches

CAF-reprogramming strategies aim to transform immunosuppressive CAFs into dormant or tumor-suppressing phenotypes by delivering agents such as TGF-β inhibitors, Hedgehog pathway inhibitors, or epigenetic modulators (48–50). Nano-strategies enable the site-specific and sustained delivery of these regulators within the TME, boosting effectiveness while reducing systemic side effects (51–53). This strategy offers a prospective approach to deplete CAFs by maintaining beneficial functions of stroma and alleviating tumorigenic signaling.

Nano-drug delivery systems can convert CAFs to a quiescent state by delivering modulatory molecules that inhibit their activation pathways. For example, TGF-β pathway inhibitors or microRNA-loaded drug delivery nano-systems restore a non-activated, stromal-supportive phenotype by inhibiting pro-fibrotic and immunosuppressive gene expression in CAFs (54–56). This reprogramming strategy attenuates CAF-mediated immune rejection and extracellular matrix (ECM) remodeling, thereby creating a more supportive TME for immunotherapy.

### 3.4 ECM remodeling and barrier disruption

The newly emerged nano-strategies to degrade ECM, such as collagenase-loaded nanoparticles, represent a potential approach to remodel the fibrotic stroma formed by CAFs. These nano-strategies contribute to the disruption of the physical barriers, which impede T-cell infiltration and restrict diffusion of modulatory agents into the tumor interior, by enzyme-mediated degradation of collagen and other ECM compositions (57, 58). This ECM-modulating approach can remarkably improve the efficacy of nano-immunotherapies by increasing their exposure to tumor cells. However, precise modulation is required to prevent exaggerated ECM degradation, which could potentially facilitate tumor invasion and metastatic progression (59).

### 3.5 Modulating CAFs-immune cell crosstalk

From the perspective of modulating CAF-immune cell crosstalk, targeting CAF-secreted factors, such as CXCL12, has been shown to alleviate T cell migration barriers and enhance immune cell infiltration within tumors (60). Building on this approach, various nano-delivery platforms have been developed to co-deliver CAF inhibitors and immune checkpoint blockers (ICBs), enabling synergistic effects through CAF reprogramming and immune activation, thereby improving the overall efficacy of immunotherapy.

### 3.6 Synergistic effects with immunotherapy

Combining CAF-targeting nanoplatforms with ICIs has emerged as a promising strategy to overcome the immunosuppressive tumor microenvironment and enhance therapeutic efficacy (61). By disrupting CAFs-mediated signaling, these nanoplatforms can enhance antigen presentation by dendritic cells and promote the activation and infiltration of cytotoxic T lymphocytes. In parallel, modulation of CAFs activity leads to a reduction in immunosuppressive cell populations such as myeloid-derived suppressor cells (MDSCs) and regulatory T cells (Tregs), further relieving immune suppression. This synergistic approach reprograms both the stromal and immune components of the tumor microenvironment, paving the way for more durable and effective responses to ICIs (62, 63). Examples of combining CAF-targeting nanoplatforms and immunotherapy are shown in **Table 1**.

**Table 1:** Representative nano-platforms of combined targeting CAFs and immunotherapy.

Nanoparticles	CAFs targeting methods	Combating CAF strategies	Immunotherapeutics	Tumor models	Reference
lipid nanoparticles (LNPs)	FAP-2286	Blocking both glycolysis and mitochondrial respiration in CAFs	αPD-1	Orthotopic RM-1 tumor model	(68)
Micelles	fibronectin-targeting peptide Fmoc-K(PpIX)-CREKA	ECM remodeling and CAFs reprogramming	αPD-L1	Subcutaneous 4T1 tumor model	(69)
Tumor cell-derived extracellular vesicles	No	Reprogram CAFs and reduces the tumor ECM	YM101	Subcutaneous H22 tumor model	(70)
CAF and cancer cell membrane coated mesoporous silica NP	Homotypic targeting CAFs	Reprogram abnormal VB3 metabolism in CAFs	CAF and cancer cell membrane	Subcutaneous 4T1 tumor model	(71)
Dendrimers	Passive targeting of EPR effect	ECM degradation by ECM-specific enzymes	αPD-L1	Subcutaneous 4T1 and MDA-MB-231 tumor models	(72)
Nanocomplexes	Aminoethyl anisamide	Genetically engineering CAFs into APCs	HSP70-initiated plasmids, including CD86 and PD-L1 trap	Orthotopic high-fibrotic 4T1 breast tumor model	(73)
Micelles	Passive targeting of the EPR effect	Anti-fibrotics relieve the immunosuppressive function of CAFs.	αPD-1 and αCTLA-4	Orthotopic 4T1 and E0771 breast tumor model	(74)
Tumor cell-derived extracellular vesicles	Passive targeting of the EPR effect	Regulate intratumoral CAFs and reduce tumor ECM	αPD-1 and TGF-β inhibitor SB431542	Subcutaneous xenograft KYSE30 tumor model	(75)
Nanoliposome	EpCAM-targeting	Repolarize CAFs into quiescent fibroblasts	αPD-1	LLC subcutaneous tumor model	(76)

Preclinical studies have demonstrated that combining CAF-targeting strategies with ICIs significantly improves antitumor responses by enhancing T cell infiltration and reducing stromal barriers. For instance, blockade of the CXCL12/CXCR4 axis using nanocarriers has been shown to sensitize tumors to anti-PD-1 therapy in murine models of pancreatic and breast cancer (64, 65). Early-phase clinical trials are also exploring this synergy; one notable example is the combination of CXCR4 inhibitors with ICIs in patients with solid tumors, showing promising signs of improved immune activation and partial responses (66, 67). These findings support the translational potential of CAF-targeted approaches in amplifying the therapeutic benefits of current immunotherapies.

## 4 Translational potential and current challenges

Despite the promising synergistic outcomes of CAF-targeting nano-strategies combined with immunotherapy in preclinical settings, several translational challenges remain. First, significant disparities exist between murine models and human tumors, particularly in terms of CAF composition, immune cell profiles, and stromal architecture, which may lead to inconsistent therapeutic responses (77, 78). The intrinsic heterogeneity of CAFs further complicates effective targeting, as diverse CAF subpopulations (e.g., iCAFs, myCAFs, apCAFs) may play opposing roles in tumor progression and immune modulation (79).

Moreover, nanocarrier systems encounter biological barriers *in vivo*, including limited tumor penetration, off-target distribution, potential immunogenicity, and suboptimal pharmacokinetics. Ensuring the efficient and safe delivery of therapeutic agents to CAF-rich tumor sites remains a major hurdle (80). From a translational perspective, regulatory challenges and scalability issues further impede the clinical adoption of CAF-targeted nanomedicines. Concerns regarding long-term toxicity, manufacturing reproducibility, and quality control must be addressed to enable industrialization (81).

Nevertheless, several clinical trials are underway to evaluate CAF-targeted therapies in combination with immune checkpoint blockade. Notably, FAP-targeted nanoparticles and radiopharmaceuticals are currently being tested in solid tumors, providing early evidence of safety and potential immune modulation (82, 83). These trials mark an encouraging step toward validating CAF-targeted nano-strategies in the clinic.

## 5 Conclusion and outlook

CAFs play a central role in shaping the immunosuppressive tumor microenvironment and driving resistance to immunotherapy. Targeting CAFs has thus emerged as a promising strategy to overcome the limitations of current immunotherapeutic approaches. In recent years, nanotechnology-based platforms have demonstrated outstanding potential in modulating CAFs’ functions, offering precise delivery, controlled release, and multifunctional integration to reshape immune dynamics and enhance therapeutic responses. Particularly when combined with ICIs. These nano-strategies can simultaneously suppress CAF activity, promote T cell infiltration, and boost immune activation, leading to synergistic antitumor effects. However, the intrinsic heterogeneity and plasticity of CAFs pose significant challenges to targeted therapy, while issues associated with the *in vivo* delivery efficiency, biosafety, and immunogenicity of nanocarriers remain unresolved. Moving forward, deeper mechanistic insights into CAF-immune interactions and subtype-specific functions are urgently needed, along with the development of standardized translational and clinical evaluation frameworks. With continued innovation, CAF-targeted nano-immunotherapies hold great promise as customizable and translatable platforms, potentially transforming the treatment landscape for solid tumors.
